# The effect of 12-weeks Nutritional supplementation on Nutritional Intake and Status among Indonesian Older Outpatients with Malnutrition Risk, the Prolansia study: a randomized controlled trial

**DOI:** 10.1016/j.jnha.2025.100548

**Published:** 2025-03-27

**Authors:** Esthika Dewiasty, Sjors Verlaan, Rahmi Istanti, Fariza Rahmah, Eugene Satryo, Lisette CPGM de Groot, Siti Setiati

**Affiliations:** a Division of Human Nutrition and Health, Wageningen University, Wageningen, The Netherlands; b Division of Geriatric Medicine, Department of Internal Medicine – Faculty of Medicine, Universitas Indonesia, Cipto Mangunkusumo Hospital, Jakarta, Indonesia; c Universitas Indonesia Hospital (RSUI), Depok, Indonesia; d Department of Nutrition and Dietetics, Faculty of Health, Sport and Physical Activity, Amsterdam University of Applied Sciences, The Netherlands; e Department of Operating Rooms, Radboud University Medical Center, Nijmegen, The Netherlands; f Center of Clinical Epidemiology and Evidence-Based Medicine, Cipto Mangunkusumo Hospital – Faculty of Medicine, Universitas Indonesia, Jakarta, Indonesia; g Faculty of Health Sciences, Universitas Muhammadiyah Prof. Dr. Hamka, Jakarta, Indonesia; h Faculty of Medicine, Universitas Indonesia, Jakarta, Indonesia

**Keywords:** Oral nutrition supplementation, Malnutrition, Nutrient intakes, Older outpatients, Indonesia

## Abstract

**Background:**

Standard care for older outpatients who are at risk of malnutrition in Indonesia is still based on the 2017’s recommendations of the Indonesian Geriatrics Society. and does not provide nutritional supplementation as recommended by ESPEN guidelines 2019/2022.

**Objective:**

We compared the effects of supplementation of at least 400 kcal/day including 30 g or more of protein/day as nutritional intervention as recommended by the ESPEN Guideline with standard care recommended by The Indonesian Geriatrics Society, in Indonesian older adults who are at risk of malnutrition.

**Methods:**

Older outpatients (60 years or older) at a geriatric clinic of the national referral hospital with or at risk of malnutrition were recruited. They were randomly allocated to 12 weeks of supplementation with a nutrient dense drink twice a day on top of standard care compared to standard care only. We assessed energy and nutrient intake at baseline, after 6 and 12 weeks and nutritional status, physical performance, and vitamin D level at baseline and after 12 weeks. Data analyses were blinded.

**Results:**

As many as 105 older outpatients (65 % women, mean age 72.5 years, SD = 6.3) were randomly assigned to the intervention (n = 54) and the control group (n = 51). One hundred and one participants completed the intervention, with an average compliance of 90% to the nutritional intervention. Nutritional supplementation significantly increased daily intake of energy, protein, total fat, vitamin D, vitamin B12, calcium (all p values <0.001) and carbohydrate (p = 0.002) in both men and women after 12 weeks. The intervention group showed an increase in vitamin D levels (p = 0.008). Furthermore, the intervention group gained more body weight than the control group did (p = 0.021)), especially in women (p = 0.017). Women in the intervention group also showed more increase in skeletal muscle mass (p = 0.023). Improvements in muscle strength and physical performance were not statistically different between the groups. No significant adverse effects were noted.

**Conclusion:**

Nutritional supplementation is effective in improving nutritional intake and status among Indonesian outpatients with malnutrition risk, which has the potential to support and enhance the standard of care.

## 1 Introduction

### 1.1 Background

Indonesia has an aging population. Data provided by the Indonesian Statistics Bureau in 2023 showed that the proportion of older persons in Indonesian population amounts to 11.7%. [1]. Recently a systematic review uncovered that there is a high risk of malnutrition along with poor macronutrient and micronutrient (most profound for vitamin D and calcium) intake among Indonesian older outpatients [2]. Malnutrition may lead to various health problems, including frailty, morbidity, and mortality [3].

Supplementation might be an effective strategy according to the European Society for Clinical Nutrition and Metabolism (ESPEN) guidelines for clinical nutrition and hydration in geriatrics in 2019 and 2022, to achieve adequate energy and nutrient intake, and to maintain or improve nutritional status in older persons with malnutrition or at risk of malnutrition [4]. The ESPEN’s practical guideline also recommends that oral nutritional support (ONS) should provide at least 400 kcal/day including 30 g or more of protein/day and should at least be given for one month [5]. Thereby, maintenance or improvement of function, activity, capacity for rehabilitation and quality of life, support of independence and a reduction of morbidity and mortality is intended [6].

However, the evidence of beneficial effects of nutritional supplementation needs further strengthening. A systematic review and meta-analysis conducted by Correa-Perez et.al. found that the efficacy of non-pharmacological interventions (dietary counselling and ONS, ONS combined with exercise, nutrition delivery systems) to treat malnutrition in older persons were inconsistent, highlighting the lack of high-quality evidence to indicate which interventions are effective in treating malnutrition in older people [7]. On the other hand, two pooled analyses of individual participant data from nine RCTs conducted by Reinders et al. and van Zwienen-Pot et.al found that nutritional intervention, provided as dietary counselling combined with ONS is effective for improving energy and nutrient intake as well as body weight gain [8,9]. These reviews however lack data from Asian settings.

In Indonesia, to date, ONS for older individuals with malnutrition and those at risk, including older outpatients has not been established as a national policy, whereby standard care for older adults who are at risk of malnutrition is still based on the 2017’s recommendations of the Indonesian Geriatrics Society, which strongly recommended nutritional counselling [10].

### 1.2 Objective

The primary objective of this study aimed to compare effectiveness of standard care including nutritional counselling vs standard care including nutritional counselling and supplementation, in terms of energy and nutrient intake as well as nutritional status, in Indonesian older outpatients who are at risk of malnutrition. In detail, we investigated the effects of consuming a nutrient dense drink (NDD) twice a day for 12 weeks on energy and nutrient intake by evaluating differences in changes for this primary outcome between the intervention arm receiving the NDD and the standard care arm.

We also evaluated differences in changes for the secondary outcomes between the intervention arm receiving the NDD and the standard care arm. Our secondary outcomes of this study were nutritional status, including body weight, body composition, physical performance, vitamin D level, and non-elective hospitalization after 12 weeks of intervention.

## 2 Methods

The Prolansia study has been registered and approved by The Ethics Committee of The Faculty of Medicine Universitas Indonesia- Cipto Mangunkusumo Hospital with regards to the protection of human rights and welfare in medical research. The approval number of this study is 20-07-0768. The study was conducted according to the principles of the Declaration of Helsinki (version 2013) and has been registered at ClinicalTrials.gov with protocol ID Prolansia01 and ID number NCT06068816. All participants provided written informed consent.

### 2.1 Study design and setting

This Prolansia study was a randomized controlled trial, with two parallel intervention arms: standard care plus nutritional supplementation, versus standard care for 12 weeks. This study was conducted at the geriatric clinic and internal medicine outpatient clinics at the National Referral Hospital, Dr. Cipto Mangunkusumo Hospital Jakarta, Indonesia.

### 2.2 Participants

All patients aged 60 years and older who visited the outpatient clinics at the National Referral Hospital, Dr. Cipto Mangunkusumo Hospital Jakarta, Indonesia, in the years 2022–2023 were screened for their eligibility for participation in this study. Potentially eligible participants were screened for malnutrition or risk for malnourishment using the mini nutritional assessment full form (MNA-FF). Patients were excluded if they had an estimated glomerular filtration rate (eGFR) ≤30 ml/min per 1·73m^2^, had edema, had a poor prognosis based on the evaluation of their attending physicians (such as frailty, totally dependent, or having terminal condition of illnesses), had communication difficulties because of aphasia, dementia, or did not understand the Indonesian language, or were unwilling to participate in our study. We retrieved eGFR data from the patient’s medical record or the eGFR laboratory test. After being fully informed, patients gave explicit oral consent for participation in this study, which was recorded in the electronic patient record.

### 2.3 Assessments

#### 2.3.1 Screening

The coordinating investigator disseminated data on recruitment of research participants to all doctors in charge of acute wards at Cipto Mangunkusumo Hospital. The field investigators and research assistants looked for potential participants who were malnourished or at risk of malnutrition and informed the coordinating investigator. The coordinating investigator screened the eligibility of potential participants and informed the field investigators, who gave the patients the option to participate in the intervention study. If the patient was interested, the caretaker provided them with the filled information brochure and transferred their contact details to the researchers. If the researchers received the contact details, they immediately contacted the coordinating investigator to make an appointment within 24 h to give additional information. If the patient decided to participate, informed consent was signed and the potential participants were screened for (risk of) malnutrition by MNA short form. This assessment consisted of 6 short questions giving a score based on the form. If the screening score added up to less than 11 points participants could be included and additional forms of a medical history questionnaire as well as the MNA full form were filled out. If the subject was willing to participate and fit the inclusion criteria based on both the MNA and the questionnaire they underwent baseline measurements.

#### 2.3.2 Baseline measurements, intermediate measurement, and final measurement

Baseline measurements were performed on the potential participants. The measurements included screening for nutritional status using MNA short form, medical history, and screening for renal function using the eGFR method.

Eligible participants underwent body weight and height measurement, physical performance testing, and lean body mass measurement (BIA). They also received the food records diary. Standard care comprised nutritional counseling" and "adherence to recommend dietary counseling based on The Indonesian Geriatrics Society (PERGEMI) guidelines. The patients’ daily menu was based on nutritional counseling. On top of that, the intervention group received nutrient-dense drinks. They were instructed to consume a standardized breakfast, so they did not come fasted. If a participant was not able to come, we offered a taxi service. During follow up, participants were asked to hand in their empty packages of NDD (for the intervention group), as well as food records (for both intervention and control groups). We anticipated that participants would be at our research facility for three hours. During the visits, we offered them mineral water with some snacks (cookies, fruit, etc.), a lunch, and a transportation/incentive fee as well.

#### 2.3.3 Monitoring and compliance

The participants received the nutrient-dense drink every two weeks, until they finished 12 weeks of intervention. Every 2 weeks the participants were monitored by video call or home visits to provide them with the intervention product and monitor their general health conditions. Participants were advised to have the drinks between their meals, in addition to their usual diet. Compliance was registered by counting leftovers during the visits.

### 2.4 Interventions

All participants received standard care based on the Indonesian Geriatrics Society Guidelines on Malnutrition in Older Adults and Geriatric Patients 2017 (IGS Guidelines on Malnutrition 2017). [10] Participants were randomized into two groups: nutritional supplements on top of standard care or standard care only. The oral nutritional supplement used in this study was a NDD manufactured by FrieslandCampina, The Netherlands. The nutrient-dense drink was a prototype product specifically developed for the study, which meets the recent ESPEN guidelines for the dietary management of older malnourished patients. The nutrient-dense drink was provided as a nutrient dense powder product, containing 15 g whey protein from milk (Nutri Whey™ Isolate), 200 kcal energy, 400 IU vitamin D, 250 mg calcium per serving along with the full spectrum of other vitamins and minerals. The composition of NDD is provided as supplementary material 1. This nutrient-dense drink has been approved by the National Agency of Drug and Food Control of the Republic of Indonesia (*Badan Pengawas Obat dan Makanan, BPOM*) No.ST.05.04.43.433B.12.21.013334. S.

The vanilla flavored powder product was dissolved in water (or other liquid) and served as a shake. Everyday day the study participants in the intervention group consumed two servings of the nutrient-dense drink for 12 weeks. Participants were advised to consume their usual diet. All participants were also provided dietary counseling by a dietitian throughout the study. Compliance was registered by counting leftovers during these visits. Compliance should be at least 10 out of 14 servings per week.

Reflections on the safety of the intervention product comprised its protein, vitamin D and lactose content. According to the literature, long-term consumption of protein at 2.0 g/kg/d is safe for healthy adults, and the tolerable upper limit is 3.5 g/kg/d for well-adapted participants. [8] Moreover, participants with severe kidney insufficiency were excluded from the study. Therefore, increasing the protein intake by 30 grams of protein daily is without potential risk [11]. Two servings daily provide a daily dosage of 800 IU of Vitamin D, as generally recommended for maintaining musculoskeletal health in older adults [12]. Furthermore, six months intervention with 1–2 servings of a NDD containing 800 IU neither resulted in kidney function deterioration nor caused symptoms of vitamin D or calcium toxicity in frail older adults [13]. Lactose intolerance is common in the Indonesian older population [14]. The lactose content in the study product was 0.2 g per serving, thus 0.4 g per day, far below the recommended maximal levels of 12 g lactose per day for lactose-intolerant people. Thus, the NDD can be considered safe and subject to strict safety regulations of FrieslandCampina.

### 2.5 Standard care

Standard care was defined by dietary counseling and adherence to recommended dietary counseling, based on The Indonesian Geriatrics Society (PERGEMI) Guidelines on Malnutrition in Older Adults and Geriatric Patients 2017 [10]. According to these guidelines, healthcare providers can prevent malnutrition in older adults in a variety of ways, including:

• At each outpatient clinic visit, weigh the patient and assess for nutrition issues.
• Provide nutritious and various foods with taste and texture adjusted to the patient's preferences and abilities.
• Provide calorie-dense foods in tiny portions on a regular basis (small frequent feeding), particularly for older adults with poor appetite.
• Serve food as attractively as possible, with vibrant colours, and sliced into bite sizes, especially for those who have dementia.
• Signal possible loss of muscle mass, fat mass, or edema even if no change in body weight is found.
• Screen for cognitive function, mental status, history of falls, and polypharmacy.
• Evaluate oral cavity health and sensation of taste in all older adults as a risk factor for malnutrition.
• Be aware that some medications may result in deficiencies in some nutrients.
• Encourage older adults to eat with family or friends.
• Motivate the older adults to consume food that meets standard nutritional requirements.
• Educate older adults and their families about the negative effects of overly restrictive diets, as it can lead to malnutrition and other health problems.
• Correct false food beliefs in older adults and/or families, such as avoiding meat in cancer patients, avoiding eggs in dyslipidemia patients, or avoiding vegetables in hyperuricemia patients
• If necessary, perform further assessments to evaluate nutrient intake, or laboratory measurements, particularly for blood glucose and vitamin D levels.

According to the above mentioned guidelines, dietary counseling is included in standard care, and provided for both groups. The counseling was provided by dietitians, and all patients were advised to consume their meals according to the counseling. We also made sure that our advice aligned with the diet recommended by their doctors. The difference between the intervention group and control group was only in NDD supplementation.

### 2.6 Counseling and contacts with the study participants

Participants in group 1 (intervention group) received NDD and dietary counseling, while participants in group 2 (standard care) received dietary counseling only.

The participants were examined by attending physicians and the researchers at the outpatient clinics every 4 weeks. In between the hospital visits, the team of researchers followed them up every other day.

### 2.7 Outcomes

Our primary outcome was energy and nutrient intake. The secondary outcomes included nutritional status, body weight, body composition, physical performance, vitamin D level, and non-elective hospitalization.

#### 2.7.1 Nutritional intake

Nutrient and energy intake as primary outcome comprised the intake of energy, carbohydrate, protein, total fat, calcium, vitamin D, and vitamin B12. To assess dietary intake, all participants were asked to keep a 3-day food diary (2-week days and 1-week end-day) at baseline and week 12. On each visit, a trained dietician went over the diary with the participant. Methods of estimation of portion sizes included household measures, weight or volume, standard units and portions. A pre-formatted food diary was used. Furthermore, the participants were asked to fill out four questions on liking and satiety weekly. In this diary, they could also write down any deviations from the instructions.

Food diaries were checked and entered by a dietician. Portion sizes were converted to grams (weight values) using software NutriSurvey for Windows (copyright Dr. Juergen Erhardt-SEAMEO TROPMED Universitas Indonesia version 2007). The food intake was then converted to energy, macronutrient, and micronutrient amounts using the Indonesian food composition table (*Tabel Komposisi Pangan Indonesia*) published by the Indonesian Ministry of Health 2020 [15].

#### 2.7.2 Secondary outcomes

##### 2.7.2.1 Nutritional status

Body weight was measured using calibrated digital scales (SECA) accurate to 0.1 kg. Knee height was measured to estimate height using the Chumlea formula validated for Indonesia. Knee height measurement was conducted using a knee height caliper in a supine position [16]. Body mass index (BMI, kg/m2) was calculated as weight divided by height squared. We also measured middle upper arm circumference (MUAC) and calf circumference. Middle upper arm circumference was measured at the left arm in duplicate to the nearest 0.1 cm at a point midway between the lateral projection of the acromion process of the scapula and the inferior margin of the olecranon process of the ulna. Calf circumference was measured to the nearest 0.1 cm on the left leg with the respondent standing straight, feet 20 cm apart, body weight equally distributed on both feet, and at the level of the widest circumference of the calf [17].

During screening and after 12 weeks of intervention, the Indonesian version of the Mini Nutritional Assessment (MNA) full form was used to classify participants as well-nourished (score 24–30), at risk of malnutrition (score 17–23.5), or as malnourished (score <17). [18]

##### 2.7.2.2 Body composition

Body composition was measured by means of the 8-point bioelectrical impedance analysis (BIA) method (SECA type mBCA 525). Bioelectrical impedance analysis measured a segmental analysis of body composition to assess skeletal muscle mass in a lying position. This device was designed for medical use and had good validation compared with magnetic resonance imaging (MRI) and dual X-ray absorptiometry (DXA) [19,20]. We obtained information of skeletal muscle mass (SMM) in kilograms from the BIA device.

##### 2.7.2.3 Vitamin D level

Analysis of Vitamin D level was performed at Prodia laboratory using a chemiluminescence immunoassay (CLIA) for human serum 25-hydroxyvitaminD (25(OH)D). Venous puncture was carried out by research nurses at baseline and after 12 weeks. Blood samples were collected and preserved by EDTA, and transported to the Prodia laboratory for analysis.

##### 2.7.2.4 Physical performances

Physical performance was measured using the short physical performance battery (SPPB) and handgrip strength. The SPPB test is a combination of three components: gait speed (4-meter walk at a usual pace), chair stand test (time taken to rise five times in a row from a chair as swiftly as feasible without arm rests), and balance (feet side-by-side, semi-tandem and tandem) [21]. Each component was assessed on a scale of 0 (not possible) to 4 (highest performance), resulting in a total score of 0−12. The individual outcomes of the chair rise test (in seconds) and gait speed (in m/s) were also defined as separate secondary outcomes of physical performance. We measured hand grip strength in a sitting position with the elbow flexed at 90 degrees using a hydraulic hand dynamometer (JamarTM, Preston, Jackson, Missouri, USA). Two consecutive measures of grip strength in each hand were recorded to the nearest kg Maximum grip strength was calculated by taking the average of the highest measurement from both hands [22]. All procedures were performed by research assistants comprising two trained physicians, one nurse, and one dietitian. Anthropometric measurements and food record assessment were performed by a dietician. During the study, we involved the nurses and attending physicians to make sure that all procedures comply with the comprehensive geriatric assessment, good clinical practice (GCP), and safety procedures.

##### 2.7.2.5 Hospitalization

Hospitalization was defined as either all-cause or non-elective hospitalization.

### 2.8 Sample size

The sample-size was calculated based on the primary outcome using the formula of a hypothesis test for two population means. The trial was powered based on the INA-LACTASE study which showed mean protein intake for dairy users and non-dairy users of 58.4 (SD 13.7) and 49.7 (SD 14.3) g/d, respectively. [23] To achieve a power of 80%, a level of significance of 0.05, and a two-sided alternate hypothesis; the minimum sample size for each group was 41 participants per group (for two groups, a total of 82 participants). To anticipate a non-compliance rate of 25% and a dropout of 20%, the minimum sample size of our study based on anticipated non-compliance and drop out percentage were 98 and 103 participants, respectively. A final sample size of 105 participants was chosen.

### 2.9 Randomization

Participants were randomized to either receiving standard care only or standard care plus NDD. Randomization was performed using permuted blocks of 4 participants, in a 1:1 ratio to intervention or standard supplement, conducted by an independent party (The Research Coordination Center Department of Internal Medicine, Universitas Indonesia) who was not involved in the study.

### 2.10 Blinding

Due to the different treatments of both groups, it was not possible to apply a double-blind design for this study. Data analyses were performed in a blinded manner, while the assessment of outcomes such as energy, and nutrient intake, body weight, body composition, and physical performance, were performed by blinded raters.

### 2.11 Drop out criteria

All participants were followed up at six weeks and twelve weeks of the intervention period and were categorized as drop out if they refused to take end-line measurements, passed away, had severe adverse effect due to the intervention product, were lost to follow-up, or were unwilling to continue participation due to any reasons.

### 2.12 Follow-up of participants withdrawn from treatment

Participants who withdrew were not replaced by another participant and were asked to visit the research facility for endline measurements.

## 3 Statistical methods

Data were analysed using the intention-to-treat method. All data were checked for normality. Normality was assessed via visual inspection and Kolmogorov-Smirnoff test. Extreme outliers, as defined by values boundaries ± 3 standard deviations in combination with box-plot checking, were omitted in the final analyses. Independent t-test, chi-square test, or Mann–Whitney U test was used to compare values at baseline between groups for the total participants, and categorized further based on sex in univariate analysis. For dietary intake, mean values of three days were used in the analyses. Mean differences between within group changes were tested for the evaluation of the intervention effect (t-test, Mann-Whitney test). We set the statistical significance at a p-value less than 0.05. Non-elective hospitalization was presented as number and percentage. All analyses were performed in IBM SPSS Statistics version 25.0 (IBM Corp.)

## 4 Results

**Fig. 1:**
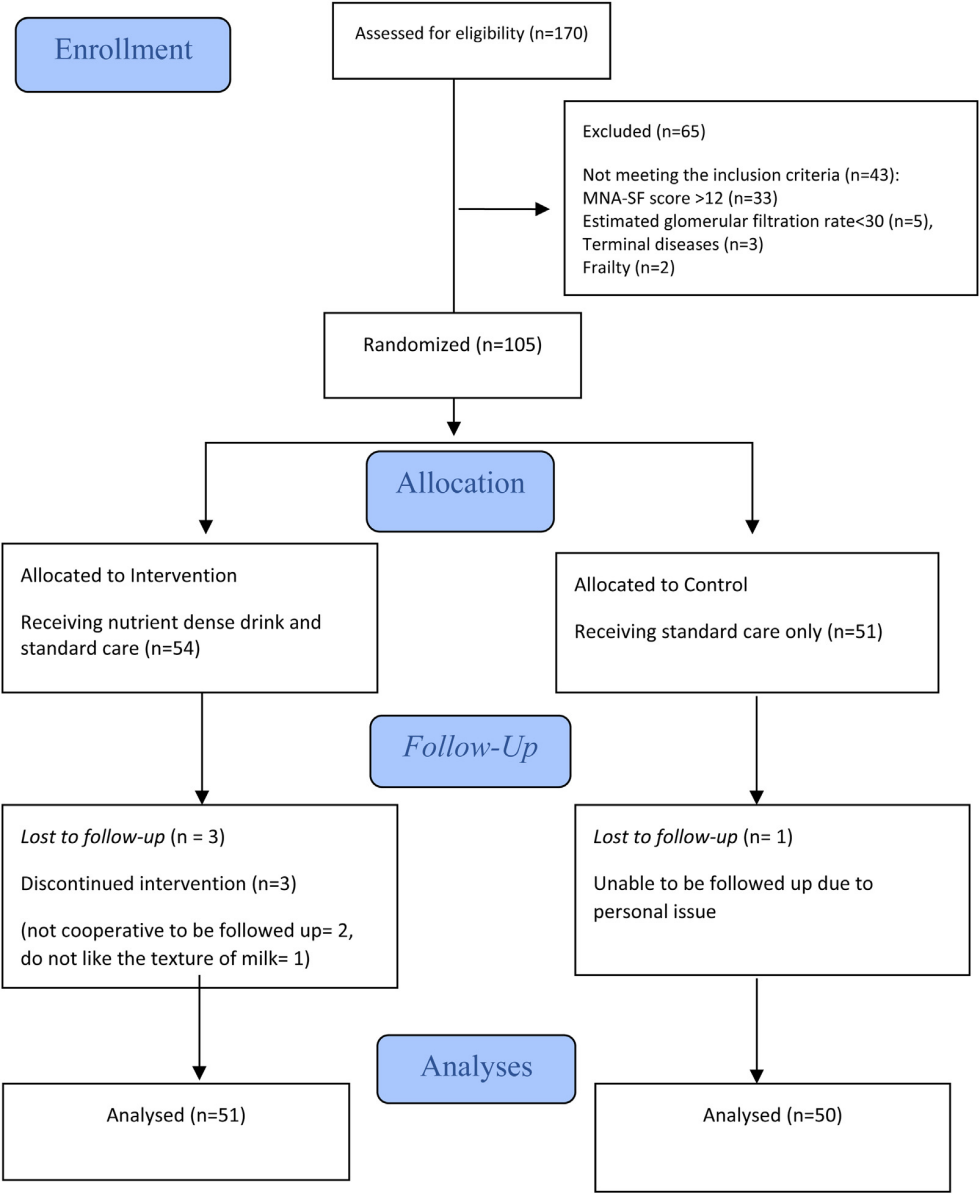
The CONSORT flow diagram of the Prolansia study.

At screening, 170 patients were eligible and underwent assessment for eligibility criteria. In total 105 patients met the eligibility criteria and were randomly assigned to the intervention (54 participants) and the control group (51 participants). At the end of the study, the intervention group consisted of 51 participants and the control group 50 participants. The dropout rate was low in both groups (intervention: 6%; control: 2%). The recruitment process was started on July, 15th 2022 and stopped on June, 9th 2023. The trial ended with the last visit of the last subject on October, 15th 2023. Fig. 1 presents the flowchart of the numbers of participants at different study stages.

**Fig. 2:**
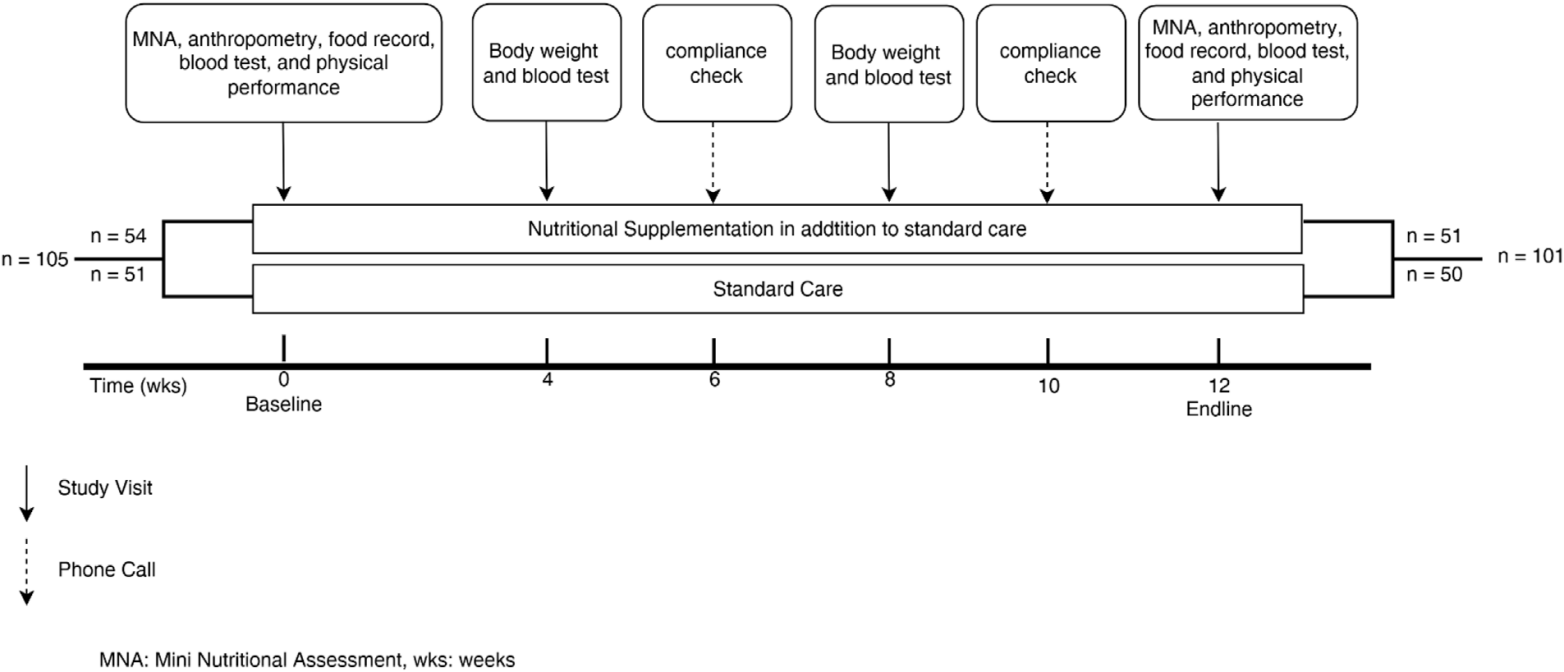
The measurement scheme of the Prolansia study.

The serial measurements of baseline characteristics and outcomes are described in Fig. 2 below.

In total, 101 participants were included in the analyses because 3 participants in the intervention group and 1 participant in the control group could not be invited for the endline measurements. Participants had a mean age of 73 (SD 7.3) years in the intervention group, and 73 (SD 5.8) years in the control group. The majority of participants were women, in both groups. Most of the study participants had chronic degenerative diseases with hypertension, osteoarthritis, and dyslipidemia as the most prevalent diseases. Baseline characteristics of the intervention and control group did not differ, except for anthropometric measurements as shown in Table 1.

**Table 1:** Baseline characteristics of study participants.

Characteristics	Group		p-value
	Intervention (n = 54)	Control (n = 51)	
Age, mean (SD)	73 (7.3)	73 (5.8)	0.621
Sex, n (%)			
Men	22 (40.7)	15 (29.4)	0.225
Women	32 (59.3)	36 (70.6)	
Educational background, n (%)			
No formal education	0 (0.0)	1 (1.9)	0.322
Elementary school	7 (13.7)	3 (5.6)	
Junior high school	4 (7.8)	3 (5.6)	
Senior high school	12 (23.5)	20 (37.0)	
University/ higher education	28 (54.9)	27 (49.1)	
Marital status, n (%)			
Married	31 (57.4)	23 (45.1)	0.107
Unmarried/ not married	7 (13.2)	3 (5.9)	
Divorce	16 (30.2)	25 (49.0)	
Caregiver, n (%)			
Yes	49 (90.7)	45 (88.2)	0.675
No	5 (9.3)	6 (11.8)	
Income, n (%)			
Below minimum income	3 (5.6)	4 (7.8)	0.639
Minimum income or higher	51 (94.4)	47 (92.2)	
Employment, n (%)			
Housewife	13 (24.1)	17 (33.3)	0.545
Retired from Government employee	17 (31.5)	18 (35.3)	
Retired from private employee	12 (22.2)	10 (19.6)	
Entrepreneur/self-employee	8 (14.8)	3 (5.9)	
Others	4 (7.4)	3 (5.9)	
History of smoking, n (%)			
Yes	3 (3.60)	1 (2.0)	0.618
No	51 (94.4)	50 (98.0)	
Functional Status			
ADL Barthel Score, mean (SD)	19.1(1.6)	19.3 (0.8)	0.453
Independent (ADL score 20)	34 (63.0)	27 (52.9)	
Mild dependent (ADL score 12−19)	20 (37.0)	24 (47.1)	0.298
Cognitive status based on MMSE score, mean (SD)	27.7(2.95)	28.1(2.9)	0.471
Comorbidities			
Hypertension	30 (57.7)	34 (68.00)	0.282
Osteoarthritis	22 (42.3)	27 (54.0)	0.237
Dyslipidemia	32 (61.5)	32 (64.0)	0.797
Diabetes mellitus	15 (28.8)	19 (38.0)	0.327
Heart failure	10 (19.2)	8 (16.0)	0.669
Hyperuricemia	8 (15.4)	8 (16.0)	0.932
Coronary heart disease	8 (15.4)	7 (14.0)	0.844
Anemia	7 (13.5)	3 (6.0)	0.205
Low back pain	4 (7.7)	5 (10.0)	0.681
Chronic obstructive pulmonary diseases	5 (9.6)	3 (6.0)	0.497
Anthropometric measurement			
Weight (kg), mean (SD)	52.3 (8.4)	56.8 (9.2)	0.010
Height (cm), mean (SD)	157.0 (7.8)	155.8 (6.9)	0.546
Waist Circumference (cm), mean (SD)	82.7 (9.3)	87.4 (9.6)	0.012
Calf Circumference (cm), mean (SD)	30.7 (2.4)	32.5 (2.8)	P<0.001
Hip Circumference (cm), mean (SD)	90.3 (6.5)	95.2 (7.2)	P<0.001
Upper arm circumference, mean (SD)	24.6 (3.2)	26.2 (3.7)	0.020
Body Mass Index, mean (SD)	21.3 (2.6)	23.4 (3.5)	0.001
Protein (g/kg BW/d), median, IQR	1.02 (0.6)	0.96 (0.48)	0.092
<0.8 gr/KgBWday	11 (20.4)	18 (35.3)	
0.8–<1 gr/KgBW/day	14 (25.9)	10 (19.6)	
1−1.2 gr/KgBW/day	8 (14.8)	10 (19.6)	
>1.2 gr/KgBW/day	21 (38.9)	13 (25.5)	

Below minimum income for Indonesian older adults: < I,800,000 IDR per month, minimum income or higher: ≥ 1,800,000 IDR per month, IDR: Indonesian Rupiah (currency), IQR: interquartile range, SD: standard deviation, ADL: activities of daily living, ASM: appendicular skeletal muscle, g: gram, g/kg BW/d: gram per kilogram bodyweight per day, IQR: interquartile range, kcal: kilocalories, mg: milligram, mcg: microgram, ng/mL: nanogram/ milliliter, MMSE: mini-mental state examination, SMI: skeletal muscle index, SPPB: short physical performance battery.

Compliance to the nutritional intervention was 90% in the group of 51 older outpatients that completed the intervention. The primary outcome energy and nutrient (carbohydrate, protein, total fat, vitamin D, calcium, and vitamin B12) intake increased statistically significantly more in the intervention group than the control group, in both within and between groups. The within groups-increases in energy intake in the total intervention group compared with control group after 12 weeks were 370 (SD 335) kcal/day and 34 (SD 344) kcal/day, respectively, with a statistically significant between group difference (336 kcal/d, p < 0.001). The protein intake increased more in the intervention group (25.6 (SD 15.6) g/day) than it did in the control group 1.7 (SD 14.6) g/day, with a between group difference amounting to 23.9 g/d (p < 0.001). The protein intake expressed per kg body weight significantly increased to 1.6 g/kg/d in the intervention group, as recommended by the ESPEN guidelines [5]. The increase in vitamin D intake in the intervention group (16.1 (SD 5.3)) was higher than that in the control group (0.1 (SD 6.2)), with a between group difference of 16,0 mcg/d (p < 0.001). The analyses results for energy and nutrient intake, stratified by sex, are presented in Table 2.

**Table 2:** Changes in energy and nutrient intake after 12 weeks of intervention.

Baseline Intake				Changes after 12 weeks						
Nutrient Intake	Intervention (n = 54)	Control (n = 51)	p-value	Intervention (n = 51)		Control (n = 50)		p-value for changes within groups after 12 weeks		p-value for differences in changes between groups after 12 weeks
Energy (kcal/d)				Intake	Delta	Intake	Delta	Intervention	Control	
Total, mean (SD)	1437 (352)	1420 (405)	0.766^a^	1807 (411)	370 (335)	1454 (379)	34 (344)	p < 0.001^c^	0.357^c^	p < 0.001^a^
Men, mean (SD)	1534 (360)	1614 (286)	0.483^a^	1915 (481)	382 (397)	1568 (220)	−46 (195)	p < 0.001^c^	0.300^c^	p < 0.001^a^
Women, mean (SD)	13164 (333)	1345 (422)	0.844^a^	1724 (333)	361 (287)	1409 (420)	65 (386)	p < 0.001^c^	0.157^c^	p < 0.001^a^
Protein (g/d)										
Total, mean (SD)	58.1 (16.0)	55.6 (20.1)	0.250^a^	84.7 (17.1)	25.6 (15.6)	57.3 (15.8)	1.74 (14.6)	p < 0.001^d^	0.403^d^	p < 0.001^a^
Men, mean (SD)	60.7 (18.9)	67.1 (16.9)	0.402^a^	89.2 (18.8)	28.6 (17.8)	64.6 (11.5)	−2.6 (18.2)	0.007^d^	0.608^d^	p < 0.001^a^
Women, mean (SD)	58.0 (13.6)	51.1 (32.2)	0.119^a^	81.3 (15.1)	23.3 (13.5)	54.5 (16.4)	3.4 (12.8)	p < 0.001^d^	0.120^d^	p < 0.001^a^
Protein intake Per Body weight per day (g/kg BW/day)										
Total, mean (SD)	1.2 (0.4)	1.0 (0.43)	0.135^a^	1.6 (0.4)	0.4 (0.3)	1.1 (0.3)	0.02 (0.27)	p < 0.001^d^	0.628^d^	<0.001^a^
Men, mean (SD)	1.1 (0.4)	1.2 (0.4)	0.693^a^	1.6 (0.5)	0.5 (0.4)	1.1 (0.3)	−0.07 (0.35)	p < 0.001^d^	0.137^d^	p < 0.001^a^
Women, mean (SD)	1.2 (0.3)	1.0 (0.4)	0.040^a^	1.6 (0.4)	0.4 (0.3)	1.0 (0.4)	0.06 (0.23)	p < 0.001^d^	0.392^d^	p < 0.001^a^
Total Fat (g/d)										
Total, mean (SD)	51.3 (17.3)	55.7 (22.8)	0.399^a^	67.5 (18.9)	16.2 (20.5)	57.5 (20.5)	1.82 (17.7)	p < 0.001^d^	0.471^d^	p < 0.001^a^
Men, mean (SD)	52.9 (18.2)	64.9 (24.0)	0.186^a^	70.9 (22.3)	17.9 (24.4)	59.6 (20.3)	−5.3 (15.8)	0.002^d^	0.233^d^	p < 0.001^a^
Women, mean (SD)	50.0 (20.1)	52.1 (21.6)	0.672^a^	65.0 (15.7)	14.9 (17.3)	56.7 (20.8)	4.6 (17.8)	p < 0.001^d^	0.132^d^	0.02^a^
Carbohydrate (g/d)										
Total, mean (SD)	187.2 (53.5)	179.6 (52.3)	0.458^a^	220.6 (67.1)	32.7 (41.3)	183.1 (52.8)	3.8 (51.3)	p < 0.001^d^	0.601^d^	0.002^a^
Men, mean (SD)	204.0 (57.4)	197.9 (47.1)	0.905^a^	234.7 (82.2)	30.7 (42.6)	199.9 (31.3)	1.2 (37.8)	0.003^d^	0.908^d^	0.04^a^
Women, mean (SD)	175.6 (50.6)	172.0 (53.6)	0.783^a^	209.8 (51.8)	34.2 (41.1)	176.9 (58.2)	4.8 (56.1)	p < 0.001^d^	0.608^d^	0.03^a^
Vitamin D (mcg/d)										
Total, mean (SD)	6.3 (5.4)	5.1 (5.0)	0.239^a^	22.4 (5.5)	16.1 (5.3)	5.1 (4.3)	0.06 (6.2)	p < 0.001^c^	0.747^c^	p < 0.001^b^
Men, mean (SD)	7.5 (6.6)	5.9 (6.2)	0.509^a^	23.9 (4.5)	16.3 (5.4)	5.4 (3.9)	−1.1 (6.5)	p < 0.001^c^	0.851^c^	p < 0.001^b^
Women, mean (SD)	5.4 (4.1)	4.8 (4.7)	0.560^a^	21.3 (6.1)	15.9 (5.3)	5.0 (4.5)	0.3 (6.1)	p < 0.001^c^	0.555^c^	p < 0.001^b^
Vitamin B12 (mcg/d)										
Total, mean (SD)	2.8 (3.1)	2.7 (3.8)	0.473^b^	4.2 (4.9)	1.3 (3.5)	2.4 (2.1)	−0.3 (4.0)	p < 0.001^c^	0.732^c^	p < 0.001^b^
Men, mean (SD)	2.5 (1.7)	3.1 (2.1)	0.333^a^	3.6 (2.8)	1.1 (1.7)	2.3 (1.6)	−0.8 (1.9)	0.001^c^	0.132^c^	p < 0.001^b^
Women, mean (SD)	3.1 (3.9)	2.5 (4.2)	0.285^b^	4.6 (6.0)	1.5 (4.5)	2.4 (2.2)	−0.1 (4.6)	0.001^c^	0.479^c^	0.011^b^
Calcium (mg/d)										
Total, mean (SD)	375 (210)	369 (215)	0.486^b^	755 (175)	379.7 (214.7)	390 (210)	21 (201)	p < 0.001^c^	0.409^c^	p < 0.001^c^
Men, mean (SD)	362 (223)	436 (159)	0.502^a^	757 (195)	395.0 (232.8)	451 (219)	16 (204)	p < 0.001^c^	0.975^c^	p < 0.001 c
Women, mean (SD)	385 (202)	343 (230)	0.170^b^	753 (161)	368.2 (203.4)	366 (205)	23 (203)	p < 0.001^c^	0.307^c^	p < 0.001 c

a p-values obtained by t- test to analyze differences in baseline and in changes between the control and intervention groups.

b p-values obtained by Mann Whitney test to analyze differences in baseline and in changes between the control and intervention groups.

c p-values obtained by Wilcoxon signed rank-test to analyze differences within groups.

d p-values obtained by dependent t-test to analyze difference within groups.

The results of secondary outcomes are shown in Table 3. The univariate analysis showed a significant effect of the nutritional intervention on nutritional status in terms of body weight change between baseline and after 12 weeks. Mean changes in the intervention group (1.1 kg (SD 1.98)) and the control group (0.2 kg (SD 1.7)) differed by 0.9 kg (p = 0.021). The difference in change was more pronounced in women (1.2 kg, p = 0.017), than in men (0.4 kg, p = 0.493). We observed a statistically significant improvement of skeletal muscle mass after 12 weeks among women after the intervention (3.0 kg, p = 0.023). We observed that the level of vitamin D increased more (2.5 ng/mL, p = 0.008) in the intervention group (1.9 ng/mL (SD 11.0)) than it did in the control group (-0.6 ng/mL (SD 4.9). No changes in muscle strength and physical performance were statistically different between groups and sexes.

**Table 3:** The Effect of Nutrient Dense Drink on Secondary Outcomes: Change (Delta) in Nutritional Status, Body Composition, Physical Performance, and Vitamin D levels after 12 weeks.

Variable	Baseline		Changes (delta) after 12 weeks		*p-value for changes*
	Intervention (n = 54)	Control (n = 51)	Intervention (n = 51)	Control (n = 50)	
Weight, Kg					
Total, mean, (SD)	52.6(8.4)	56.9(9.2)	1.1 (2.0)	0.2(1.7)	0.021^b^
Men, mean (SD)	57.2 (7.5)	60.6 (9.2)	0.7 (1.6)	0.3 (1.4)	0.493^a^
Women, mean (SD)	49.1 (7.4)	55.3 (8.9)	1.4 (2.3)	0.2 (1.9)	0.017^a^
MNA Score					
Total, mean (SD)	20 (2.2)	21 (2.5)	4.6 (3.3)	3.6 (3.4)	0.201^a^
Men, mean (SD)	20 (2.5)	22 (1.7)	3.3 (2.2)	3.8 (3.6)	0.617^b^
Women, mean (SD)	20 (2.0)	21 (2.7)	5.5 (2.9)	3.8 (3.9)	0.130^a^
Body Composition					
Skeletal Muscle Mass (kg)					
Total, mean (SD)	16.0 (4.8)	17.0 (5.6)	1.6 (7.0)	0.2 (6.4)	0.158^b^
Men, mean (SD)	20.2 (3.8)	21.3 (4.9)	−0.4 (3.5)	0.5 (2.9)	0.707^b^
Women, mean (SD)	12.7 (2.2)	15.4 (5.1)	3.3 (8.8)	0.3 (7.4)	0.023^b^
Physical Performance					
Handgrip Strength, Kg					
Total, mean (SD)	22.9 (6.6)	22.5 (6.7)	0.49 (4.6)	0.1 (4.2)	0.405^b^
Men, mean (SD)	27.1 (6.8)	28.3 (6.7)	0.2 (5.5)	0.1 (3.4)	0.982^a^
Women, mean (SD)	19.8 (4.3)	20.1 (5.2)	0.8 (3.5)	0.2 (4.6)	0.619^a^
Gait Speed, sec					
Total, mean (SD)	5.9 (3.0)	5.6 (2.9)	−0.5 (1.7)	−0.8 (4.5)	0.295^b^
Men, mean (SD)	6.4 (3.8)	5.3 (3.2)	−0.7 (2.2)	−0.7 (1.3)	0.489^b^
Women, mean (SD)	5.5 (2.3)	5.7 (2.8)	−0.3 (1.2)	−0.9 (1.3)	0.432^b^
Chair rise test, sec					
Total, mean (SD)	13.1 (5.8)	13.4 (5.7)	−1.2 (3.9)	−1.8 (3.7)	0.667^b^
Men, mean (SD)	12.7 6.1)	13.5 (6.1)	−0.5 (5.1)	−3.8 (4.99)	0.110^b^
Women, mean (SD)	13.4 (5.6)	13.3 (5.7)	−1.8 (2.8)	−1.2 (2.9)	0.423^a^
SPPB Score					
Total, mean (SD)	9.1 (2.3)	9.2 (2.3)	0.5 (1.5)	0.5 (1.5)	0.994^b^
Men, mean (SD)	9.2 (2.4)	9.2 (2.7)	0.8 (0.5)	0.1 (1.7)	0.071^b^
Women, mean (SD	9.0 (2.3)	9.3 (2.2)	0.3 (1.5)	0.7 (1.5)	0.262^b^
Biochemical Measurement					
Vitamin D level, ng/mL)					
Total, mean (SD)	28.7 (14.7)	26.5 (11.4)	1.9 (11.0)	−0.6 (4.9)	0.008^b^
Men, mean (SD)	27.2 (7.5)	30.1 (9.7)	4.12 (8.2)	−1.6 (4.3)	0.009^b^
Women, mean (SD)	29.9 (18.4)	25.1 (11.8)	0.3 (12.6)	−0.3 (5.1)	0.262^b^

IQR: interquartile range, kg: kilogram, kg/m2: kilogram per meter square, ng/mL: nanogram/milliliter, SD: standard deviation SPPB: short physical performance battery.

a p-value by t-test to analyze differences between the control and intervention groups.

b p-value by Mann–Whitney test to analyze differences between the control and intervention groups.

During the study, we had two hospitalizations for both the intervention and control group, which resulted in no effect. There were two participants in the intervention group who were hospitalized, the first one was hospitalized due to acute complications of diabetes mellitus, and another one was caused by elective surgery. In the control group there were two participants who were hospitalized due to elective surgery. Except for hospitalization, there were no adverse events (AEs) and serious adverse events (SAEs) in both groups. All hospitalization cases were reviewed by the Ethics Committee and concluded that the cases were not related to the study products.

## 5 Discussion

This study showed that 12 weeks of nutritional supplementation had a significant effect on energy, nutrient intakes, and nutritional status in older outpatients with (or at risk of) malnutrition. We observed significant body weight and skeletal muscle mass gain among women in the intervention group. Our nutritional intervention did not lead to statistically significant differences in physical performance.

The unique aspect of this study is the NDD used in this study, being a specifically developed prototype product. Its nutritional composition meets recent ESPEN guidelines for dietary management of older malnourished patients. With minimal lactose levels, adverse effects of consuming the NDD were minor. Thereby, consumption of this NDD was suitable and safe to correct inadequacies of nutrient intakes in the general Indonesian older adults population, as reported by a multicenter study by Setiati et al., [23] and a previous cross-sectional study with the same source population [24]. Application of a dairy-based nutritional intervention in Indonesia and its effectiveness, considering the high prevalence of lactose intolerance in older adults (66%) and low milk consumption culture as compared to Western and other Asian countries, becomes a point of contention [14].

Improvements of energy and macronutrients- particularly protein-, vitamin D, vitamin B12, and calcium intake after 12 weeks of intervention with NDD support the ESPEN recommendations on clinical nutrition and hydration in geriatrics, stating that older persons with malnutrition or at risk of malnutrition with chronic conditions shall be offered ONS when dietary counselling and food fortification are not sufficient to increase dietary intake and reach nutritional goals [4]. The nutritional supplementation provided in this study as two servings of NDD per day, per serving contained 15 g protein, 200 kcal energy, 400 IU vitamin D, 250 mg calcium, and a full spectrum of the other vitamins and minerals. In combination with standard intervention for nutritional care in Indonesia, the supplementation served as NDD significantly improved daily energy and nutrient intake. While some large intervention studies in Western populations and some in Asian populations showed that dairy-based nutritional supplementation improves nutrient intake and health outcomes [25,26]; we found limited data reporting on the effect of a similar intervention in Indonesian older adults. The supplementation might address the problem of poor macronutrient and micronutrient intakes, most notably for protein, calcium, vitamin D, and vitamin B12 among Indonesian older adults [2]. The results for our primary outcome are consistent with the results of a pooled analysis of individual participant data from nine RCTs, as part of the Joint Program Initiative Malnutrition in the Elderly (MaNuEL) Knowledge Hub, which reveals that nutritional interventions have a positive effect on energy intake and body weight. Furthermore, the study concluded that dietary counselling combined with ONS, similar to the intervention in our study, is the most effective intervention [8]. The results of our study showed that protein intake expressed per kg body weight significantly increased to 1.47 g/kg/d in the intervention group. This finding proved that supplementation with the protein-rich NDD led to a significant increase of the older outpatients consuming more than 1.2 g/kg/d, which is suggested by the ESPEN guidelines for older people who are malnourished or at risk of malnutrition (Deutz et al. 2014) [5]. The increased protein intake may have contributed to the observed improvement in skeletal muscle mass in women. Although our nutritional supplementation succeeded in improving energy and nutrient intake, we did not find significant effect on physical performance. Our study results are in line with the results of a pooled analyses of individual participant data from nine RCTs, which concluded no effects of nutritional interventions on handgrip strength (as a proxy for muscle strength in older adults who are at risk of malnutrition) [27]. Our study results are also in line with a comparable RCT conducted in a Singaporean population which compared the effect of 6 months intervention of fortified ONS and dietary counselling vs. placebo and dietary counselling, which found no significant differences in SPPB total score [28].

Insignificant effects of our nutritional supplementation on physical performance might be explained by the baseline characteristics of our population. Compared with the PROVIDE study which had a similar intervention but different population (sarcopenic older adults with mobility issues) and worse baseline values of physical performance, their 13 weeks of nutritional supplementation found an improved chair stand time in the intervention group [25]. This comparison may imply that the magnitude of physical performance improvement elicited by nutritional supplementation may depend on how severe the baseline conditions are to begin with. In addition, physical activity, especially in the form of resistance exercise, is required as an anabolic trigger to stimulate muscle protein synthesis. These findings emphasize that physical activity, especially in the form of resistance exercise, is required as an anabolic trigger to stimulate muscle protein synthesis, resulting in improvement of skeletal muscle mass and function [29,30].

The strength of our study comprising the low drop-out number (<5%) and good compliance (≥90%), our study succeeded in delivering dairy-based nutritional intervention for older outpatients in a population with a high prevalence of lactose intolerance [14]. This supplementation is considered an appropriate interventional strategy, opening the door for more nutrient-dense dairy-based foods for Indonesian older adults.

Another strength of this study was our ability to conduct this trial in routinely visiting community-dwelling older adult outpatients during the COVID-19 pandemic. Although COVID-19 may have contributed to lowered adherence to appointments due to precautions towards infection [31], we managed this issue by applying universal caution guidelines to maintain compliance. We ensured that all procedures were performed by professional staff. Prior to the study commencement, all research assistants had been trained to comply with the good clinical practice (GCP) procedures by a certified GCP trainer. A physician specialized in physical rehabilitation also trained them to perform physical performance tests. Anthropometric measurements and food record assessment were performed by a professional dietician. During the study, we involved the nurses and attending physicians to make sure that all procedures comply to the comprehensive geriatric assessment and safety procedures. We closely monitored the participants during the study, with each field investigator having responsibility to monitor a list of participants. All participants were given the contact number of field investigators for enquiries at any time. Every 2 weeks, monitoring by video call or home visit was done to provide them with the intervention product and for health monitoring. Every 4 weeks the participants visited the study site for physical examination and outcome measurements with accordance to COVID-19 guidelines. These strategies were instrumental in maintaining compliance, resulting in the low drop-out number.

Some participants, the women, voluntarily engaged in physical exercise program after randomization. It appeared that the women might be more active than men. which might affected the body composition and physical performance [25]. This possibly explaining the pronounced effects in women for skeletal muscle mass and body weight gains. Our body composition findings may suggest that with proper physical activity, nutritional supplementation can elicit anabolism, resulting in improvement of muscle quality. This improvement might be mediated by upregulation of mitochondrial biogenic and oxidative phosphorylation in aging muscle [30].However, since the proportion of women who participated in the physical exercise program is equally distributed between control and intervention group we consider the results unbiased.

Our measurement of vitamin D levels using CLIA might not have been as accurate compared to isotope dilution-online solid phase extraction liquid chromatography-tandem mass spectrometry (IDXLC- MS/MS) as the reference test, affecting its validity. Though statistically significantly different from the control group, the increment of vitamin D level increase in the intervention group was lower than our a priori hypothesis. The NDD used in our study also contained 800 I.U/day, so we expected higher increment of vitamin D level in our study participants. A study conducted by Vaes M.M. et al. showed the changes in serum 25(OH)D3 status by treatment group throughout the 24 week intervention period. One month of supplementation 20 mcg (800 I.U) vitamin D3 already showed large differences in achieved serum 25(OH)D3 levels, with a mean of 34 nmol/L (equal to13.6 ng/mL) [32]. As a result, the efficacy of nutritional intervention on vitamin D level in our study was inconclusive. Although a three-day food records, including two days during the weekdays and one day during the weekend, is generally considered sufficient to asses variability of dietary intake for each subject; no further adjustment for intra-individual variability was applied, which might be considered a limitation.

In conclusion, nutritional supplementation served as NDD for 12 weeks in addition to standard care is safe and effective to increase energy, macronutrient, and almost all essential micronutrient intakes for Indonesian older adults. The intervention also improved nutritional status, particularly body weight. Future trials should investigate the effect of combined nutritional intervention and resistance physical exercise to improve nutritional status and physical performance. We recommend nutritional supplementation for the older outpatient population, as a population at risk of malnutrition, to be included in the Indonesian guidelines for nutritional intervention for the older adults population.

## CRediT authorship contribution statement

**ED**: Conceptualization, data curation, formal analysis, investigation, methodology, project administration, resources, supervision, validation, visualization, writing – original draft, writing – review & editing. **SV**: Conceptualization, data curation, funding acquisition, investigation, methodology, supervision, validation, writing – original draft, writing – review & editing. **RI**: Data curation, project administration, formal analysis, resources. **FR**: Formal analysis, investigation, resources, writing – original draft. **ES**: Data curation, formal analysis, methodology, writing – review & editing. **LdG:** Conceptualization, funding acquisition, investigation, methodology, resources, supervision, validation, writing – original draft, writing – review & editing. **SS**: Conceptualization, data curation, investigation, methodology, resources, supervision, validation, writing – original draft, writing – review & editing

## Sources of funding and other support

The Prolansia study was funded by The Judith Zwartz Foundation, The Netherlands, and supported by The FrieslandCampina Netherlands (FC-NL) which produced, prepared and labeled the nutrient dense drink products according to their safety standards. They provided the products with the necessary certificates regarding composition and suitability for human consumption (based on the microbial tests) within the specified expiration date. Products were delivered to the test site by FC-NL and were stored under the recommended conditions. The design, production, labeling, and delivery of the study products were performed by FrieslandCampina the Netherlands. The sponsors had no role in execution of the experiments, nor in statistical analyses or interpretation of results.

## Declaration of competing interest

The authors declare that they have no known competing financial interests or personal relationships that could have appeared to influence the work reported in this paper.
