# Supplementary material for: The effect of 12-weeks Nutritional supplementation on Nutritional Intake and Status among Indonesian Older Outpatients with Malnutrition Risk, the Prolansia study: a randomized controlled trial
Source: J Nutr Health Aging. 2025 Mar 27;29(6):100548. doi: 10.1016/j.jnha.2025.100548 (PMC12173013; doi:10.1016/j.jnha.2025.100548)
Supplement: Supplementary file 1 [file mmc1.doc]

**Supplementary data 1. Nutritional composition of the Prolansia Nutrient Dense Drink**

|  | **Per serving**  **(47 g powder & 100ml water)** | **Per 100 g powder** |
| --- | --- | --- |
| Energy, kcal | 200 | 418 |
| Protein (whey protein and peptides), g | 15,0 | 32,1 |
| total fat , g | 7,8 | 16,7 |
| Carbohydrates, g  Sugars  Lactose | 17  6.3  0.2 | 37.4  13.6  0.4 |
| Fibers, g | 0 | 0 |
| Vitamin D, mcg | 10 | 21.4 |
| Vitamin D3, IU | 400 | 857 |
| Vitamin A,mcg | 153 | 328 |
| Vitamin K, mcg | 26.0 | 55.7 |
| Vitamin C, mg | 17.3 | 36.9 |
| Thiamin, mg | 0.2 | 0.5 |
| Riboflavin, mg | 0.2 | 0.5 |
| Vitamin B6, mg | 0.3 | 0.6 |
| Niacin, mg EN | 2.6 | 5.7 |
| Folic acid, mcg | 72 | 154 |
| Vitamin B12, mcg | 0.4 | 0.9 |
| Panthotenic acid, mg | 0.9 | 1.8 |
| Biotin, mcg | 6.9 | 14.8 |
| Vitamin E, mg a-TE | 2.3 | 4.9 |
| Sodium, mg | 38 | 82 |
| Chloride,mg | 231 | 495 |
| Potassium, mg | 360 | 771 |
| Calcium, mg | 250 | 535 |
| Phosporus, mg | 144 | 308 |
| Magnesium, mg | 37 | 79 |
| Iron, mg | 1.8 | 3.9 |
| Zinc, mg | 1.9 | 4.0 |
| Copper, mcg | 135 | 289 |
| Iodine, mcg | 23.0 | 49.3 |
| Selenium, mcg | 9.0 | 19.3 |
| Manganese, mg | 0.7 | 1.4 |
