# Supplementary material for: The effect of 12-weeks Nutritional supplementation on Nutritional Intake and Status among Indonesian Older Outpatients with Malnutrition Risk, the Prolansia study: a randomized controlled trial
Source: J Nutr Health Aging. 2025 Mar 27;29(6):100548. doi: 10.1016/j.jnha.2025.100548 (PMC12173013; doi:10.1016/j.jnha.2025.100548)
Supplement: Supplementary file 2 [file mmc2.doc]

**Supplementary data 2. List of Abbreviation**

| **Abbreviation** | **Definition** |
| --- | --- |
| ADL | Activity of Daily Living |
| AE | Adverse Event |
| ASM | Appendicular Skeletal Muscle |
| BIA | Bioelectrical Impedance Analyzer |
| BMI | Body Mass Index |
| BW | Body Weight |
| CLIA | Chemilumunescence Immunoassay |
| Cr | Creatinine |
| DXA | Dual X-ray Absorptiometry |
| ESPEN | Europian Society for Clinical Nutrition and Metabolism |
| eGFR | Estimated Glomerular Filtratuon Rate |
| FC-NL | Friesland Campina Netherlands |
| GCP | Good Clinical Practice |
| IDXLC-MS | Isotope Dilution Online Solid Phase Liquid Chromatography-Tandem Mass Spectometry |
| IGS | Indonesian Geriatrics Society |
| IQR | Interquartile Range |
| MMSE | Mini Mental State Examination |
| MUAC | Middle Upper Arm Circumference |
| MNA-FF | Mini Nutritional Assessment Full Form |
| MNA-SF | Mini Nutritional Assessment Short Form |
| MRI | Magnetic Resonance Imaging |
| NDD | Nutrient Dense Drink |
| ONS | Oral Nutritional Support |
| PERGEMI | The Indonesian Geriatrics Society |
| RCT | Randomized Control Trial |
| SAE | Serious Adverse Event |
| SD | Standard Deviation |
| SMM | Skeletal Muscle Mass |
| SMI | Skeletal Muscle Index |
| SPPB | Short Physical Performance Battery |
